# Investigating synovial trace elements as diagnostic markers in acute septic arthritis: an exploratory study

**DOI:** 10.1007/s10067-026-08171-2

**Published:** 2026-05-22

**Authors:** Alexia Leloix, Martine Ropert-Bouchet, Théau Cavillon, Marie-Laure Island, Olivier Loreal, Pascal Guggenbuhl, François Robin

**Affiliations:** 1 UMR, InstitutNuMeCan (Nutrition Metabolisms and Cancer), Univ Rennes, INSERM, INRAE, CHU Rennes, 1317 1341 Rennes, France; 2 Rheumatology Department, Rennes University Hospital, 16 Boulevard de Bulgarie, 35200 Rennes, France; 3 Elemental Analysis and Metal Metabolism (AEM2) Platform, University and CHU of Rennes, Rennes, France

**Keywords:** ICP-MS, Septic arthritis, Synovial fluid, Trace elements

## Abstract

**Objectives:**

Acute arthritis requires a precise and rapid diagnosis, particularly to rule out an infectious cause, which constitutes a rheumatologic emergency. Cytological, bacteriological, and microcrystal analyses do not always clearly differentiate causes. We hypothesized that variation in metal concentrations in synovial fluid could provide additional clues for etiological orientation. Our objective was to evaluate their diagnostic value in acute arthritis.

**Methods:**

We conducted a retrospective, single-center study (Rennes University Hospital) using synovial fluids from the SYNOLACTATE-PLUS cohort, involving patients with acute arthritis (< 30 days). Metal quantification was performed by ICP-MS on frozen, centrifuged synovial samples. Diagnostic performance was assessed using univariate logistic regression and area under the curve (AUC).

**Results:**

Between 2019 and 2020, 105 patients (63.8% male; mean age 62.1 ± 17.1 years) were included. Monoarthritis predominated (66.7%), especially in the knee (78%). Two diagnostic groups were analyzed: septic (*n* = 8) and non-septic (*n* = 97). Iron and zinc levels were significantly higher in septic arthritis (iron: 84.2 µg/dl vs 44.0; zinc: 20.4 µmol/l vs 10.6; both *p* < 0.005). Copper and manganese were also elevated (*p* < 0.05), while strontium was decreased (29.4 vs 51.2 µg/l; *p* < 0.05). AUCs were 0.835 for iron, 0.825 for zinc, and 0.769 for manganese (*p* < 0.001). Sensitivity analysis showed high odds ratios: 27.0 for zinc/strontium ratio, 16.4 for zinc, and 13.0 for copper and manganese.

**Conclusion:**

Our results suggest that synovial metallomic profiling, especially iron, zinc, copper, manganese, and strontium, could improve etiological diagnosis of acute arthritis. Prospective validation is required.

**Table:** 

**Key Points** • *Synovial fluid metal profile varies with the etiology of acute arthritis* • *Fe, Cu, Zn, Mn, Mg, Se, and Rb increase, while Sr decreases in septic arthritis* • *A prospective study is needed to confirm these preliminary findings*

**Supplementary Information:**

The online version contains supplementary material available at 10.1007/s10067-026-08171-2.

## Introduction

Acute arthritis and joint effusion, a frequent and challenging condition, can be caused by several diseases. Joint septic location is the first diagnostic to investigate because of the high risk of chondrolysis, the potential systemic bacterial dissemination, and the high rate of death [1]. Classically, clinical presentation is monoarticular, with only 5 to 10% affecting more than one joint location. Septic arthritis can be located in all joints with a predominance for the knee, hip, and shoulder [2].

The main differential diagnosis are crystal-induced arthropathies: gout or calcium pyrophosphate deposition disease (CPPD). These causes represent the majority of acute arthritis with a clinical prevalence of 0.38 to 3.9% and a radiographic prevalence of 4% [3]. They are due to synovial crystals deposition leading to painful crises with polymorphic clinical presentations. Finally, inflammatory rheumatic diseases (IRD), including rheumatoid arthritis (RA) or psoriatic arthritis, can be responsible for acute arthritis. The diagnosis is often achieved through a complete clinical investigation with the research of target-organ damages, specific antibodies or positive status for HLAB27. X-ray analysis can be useful but mainly at later stages to characterize these chronic diseases.

Analysis of the synovial fluid (SF) is the main clue to the diagnosis in association with clinical and biological arguments to differentiate these causes. Even though diagnosing the cause of joint swelling is sometimes easy, 16 to 36% of cases remain undiagnosed [4]. Even more, the delay in diagnosis can have deleterious consequences for the patient care and for short-, medium-, and long-term evolution [5]. In case of septic arthritis, a rapid initiation of appropriate antibiotherapy can prevent septic diffusion, local chondrolysis and/or subchondral bone damage leading to surgery [5]. It can also limit hospitalization costs. These observations support the need to develop innovative approaches for a faster and more accurate diagnosis.

Actually, SF analysis consists routinely in synovial cell count leading to a rapid differentiation between inflammatory and mechanical joint effusion. Bacteriological and crystal research are also useful to diagnose septic or crystal-induced arthritis. Available diagnostic tools are limited, especially in terms of sensitivity with Gram staining yielding only 27–65% [6, 7]. Further tests can be necessary to precise the diagnosis, depending on the suspected cause. In case of septic arthritis, most developments have been made to improve and accelerate the treatment, either by identifying the bacteria [8], using biochemical markers [9], spectroscopy [10], or using composite scores [11]. However, despite this, some diagnoses remain difficult. Therefore, exploring new diagnostic tools such as trace elements assessment in SF could provide additional discriminating value.

Trace element profiling may offer a promising complementary approach for enhancing the diagnostic of acute arthritis. Indeed, metals, essential cofactors for most enzymes, vary in normal condition but also in case of inflammatory and septic conditions [12]. Trace elements have already been studied in the rheumatic field, showing, for instance, lower plasma zinc and selenium and higher copper levels in rheumatoid arthritis (RA) patients compared to healthy controls [13, 14]. In crystal-induced diseases, the increase in the plasma copper/zinc ratio could also be correlated with the onset of CPPD and gout in the general population [15, 16]. More recently, the role of metals in the pathogenesis of osteoporosis has been studied with the identification of metals associated to fracture risk. If most studies focused on plasma levels, few have investigated their concentrations in SF, mainly for pathophysiological purposes. SF of RA patients was described as highly composed of copper and zinc with poor strontium and selenium concentration compared to differential diagnosis such as osteoarthritis. Results regarding iron concentration in SF of RA patients were inconsistent [17, 18], despite positive Perls staining in synovial tissue. In osteoarthritic knee-joint effusions, Krachel et al. [19] highlighted a lower concentration of trace elements in SF than in correspondent serum.

Metallomic profiles have been studied in various septic contexts such as urinary tract infection [20], pneumonia [21] but also specific parasite contamination [22]. They are useful diagnostic tools in serum or in other biological fluids such as bronchoalveolar lavage [23, 24], urine [25], oral fluid [26], and can also support a favorable treatment outcome [27]. Thus, metallomic profiles in septic conditions are promising and support the interest of investigating them in synovial fluid.

Our work is based on the monocentric SYNOLACTATE-PLUS cohort, following the SYNOLACTATE cohort [11] composed of patients with acute arthritis evolving for less than 30 days from the rheumatology department of the Rennes University Hospital. The aim of this study was to quantify metals in the SF in order to evaluate if metals screening could be useful for diagnosis in a cohort of acute arthritis.

## Material and methods

### Study design and population

We performed a retrospective monocentric study in the rheumatology department of the Rennes University hospital, France. Synovial fluids analyzed came from the SYNOLACTATE-PLUS cohort, including adult patients with acute synovial effusion, with an evolution for less than 30 days, of native and atraumatic joints. Patients were excluded if they were under 18 years old or legally protected adults, if the progression exceeded 1 month or if they had local joint arthroplasty or intra-articular material.

We collected medical history including any previous history of known IRD, long-term treatment such as biotherapy (biologic disease-modifying anti-rheumatic drug) or conventional (conventional synthetic disease-modifying anti-rheumatic drug) treatment, antibiotics treatment within the previous 15 days and non-steroidal anti-inflammatory drugs (NSAIDs) or corticosteroid within the last 48 h. Other clinical data were collected including the duration of symptoms, localization of joint effusion, presence of fever and context of immunosuppression. The approval for this study was obtained from our institution’s ethical committee (Avis 19–8) and all patients were informed of the aim of the study and gave oral consent.

### Study protocol and measurements

Synovial fluids (SF) were obtained by arthrocentesis under strictly aseptic conditions in the rheumatology department.

Routine tests were performed immediately in the synovial fluid. White blood cell count (WBC) including the leucocyte count (/mm3) and percentage of polynuclear neutrophil cells was assessed microscopically at the laboratory. Culture with gram staining and blood culture (Bactec/PedPlus®) with 1 ml of SF, blood culture (Bactec/PedPlus®) with 5 ml of SF if possible were also performed. Crystal research and analysis were performed in the rheumatology and the anatomical pathology departments. Plasma C-reactive protein (CRP) was measured using an immunoturbidimetric assay.

Synovial fluids were stored at –80 °C after freezing. After thawing and centrifugation, the analysis of the metals of interest was performed by inductively coupled plasma mass spectrometry (ICP-MS) in the AEM2 platform (University hospital—Rennes). ICP-MS is the reference technique in multielement analysis including metal and non-metal using the sequential or simultaneous quantification of elements with a high sensitivity and a wide linear dynamic range. Although it has been successfully used to measure trace elements in serum, its application to synovial fluid remains limited [12, 19, 28].

### Diagnosis and cases definitions in the study

Septic arthritis of a native joint (SANJ) was defined according to Newman’s criteria and SF analysis when one of the following results was found: (1) pathogen was isolated from synovial fluid (Newman A criteria); (2) pathogen was isolated from blood culture with typical clinical presentation for SANJ (Newman B criteria); and (3) arthrocentesis revealed purulent synovial fluid with combination of absence of crystals and typical clinical history and absence of other diagnoses (Newman C criteria). Some samples were considered contaminated when the clinical presentation did not match to septic cause, with favorable evolution without antibiotic therapy. The final diagnosis was assessed by the Rheumatologist, considering all available data collected and verified according to the definitions cited above.

### Statistical analysis

Statistical analysis was performed with Prism 8 and R software. The Gaussian distribution of quantitative variables was verified by the Shapiro–Wilk and Kolmogorov–Smirnov test. Other quantitative variables were expressed as mean ± 95% confidence interval (CI) and the comparison of the variables was performed with Mann–Whitney test. The qualitative variables were expressed in absolute number and percentage and their comparisons were performed with the Fisher exact test.

Diagnosis performance was performed using for receiver operating characteristic (ROC) curves analysis for each metal using continuous variables with area under the curve (AUC).

Sensitivity analyses were performed based on data from ROC curves. Thresholds were determined to optimize sensitivity, balance with specificity, and according to the maximal Youden index, identifying the optimal threshold using the following calculation: Youden’s index = Sensitivity + Specificity − 1. Sensitivity (Se), Specificity (Sp), Likelihood Ratio Positive (LR +) and Likelihood Ratio Negative (LR-) were assessed. The continuous variable was transformed into a binary variable based on a predefined threshold. Univariate logistic regression was then performed to assess the association between the binary variable and the outcome, determining odds ratio (OR) and their significance (*p*-value < 0.05).

## Results

### Demographic and general data

**Fig. 1:**
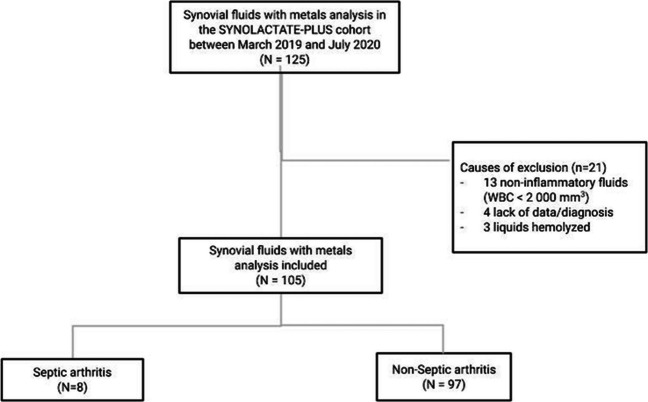
Flowchart of the study

The flowchart is presented in Fig. 1**.** Between 2019 and 2021, metals concentrations were measured in 125 synovial fluids from the SYNOLACTATE PLUS cohort. Twenty samples were excluded: 13 because of WBC under < 2000/mm^3^, 4 due to missing final diagnosis and 3 because they were hemolyzed, which could affect the results of metal analyses.

A total of 105 synovial fluids from different patients were included in our study. Mean age at inclusion was 62.1 ± 17.6 years, with a slight majority of men (63.8%). Arthritis were mostly monoarticular (67%), compared to oligoarticular (18.1%) and polyarticular (15.2%) forms. Joint analyzed were predominantly the knee (78%), and less frequently hip (6.6%), ankle (7.6%), or elbow (5.7%). 13.3% of patients took NSAIDs, 11.4% corticosteroids in the last 48 h, and 23.8% antibiotics in the 15 days prior to the puncture.

Septic patients (SP) (*n* = 8) were defined by Newman’s criteria. Gram-positive cocci (*Staphylococcus; Streptococcus*; *n* = 5) but also Gram-negative cocci (*Neisseria gonorrhoeae*, *n* = 1) and Gram-positive bacillus (*Corynebacterium striatum*; *n* = 1) were documented, with one undocumented arthritis considered as septic. Non-septic patients (NSP) group (*n* = 97) was composed of cases of microcrystalline (27 monosodium urate crystals, 36 calcium pyrophosphate and 6 both) and rheumatic arthritis (10 spondylarthritis, 3 rheumatoid arthritis, 1 post-infectious Lyme arthritis, and 14 undetermined inflammatory or chronic rheumatism).

Demographic data of populations are summarized in Table 1. Septic patients were significantly younger (49 ± 16.2 compared to 63.2 ± 17.4 years old for non-septic patients (*p* < 0.05)). Male patients were predominant, representing 75% and 63% of the respective groups. Fever was reported for 39% of patients, without difference between SP (37.5%) and NSP (39.2%). The duration of symptoms before diagnosis was around 6 days in the general population, similar in the groups. Concerning the arthritis characteristics, knee was prevalent (50% for SP and 80.4% NSP). Most clinical presentations were monoarticular (75% for SP and 66% for NSP) or oligoarticular (25% for SP and 17.5% for NSP). Polyarthritis was only seen in the NSP group (16.5%). The use of NSAIDs and steroids in the last 48 h, as well as the intake of antibiotics within the past 15 days, were comparable.

**Table 1:** Characteristics of the study population

Variables	General population (*n* = 105)	Septic (*n* = 8)	Non-septic (*n* = 97)	*p*-value
Age (m ± SD)	62.1 ± 17.6	49.0 ± 16.2	63.2 ± 17.4	< 0.05
Male sex (%, n)	63.8 (67)	75 (6)	62.9 (61)	*ns*
*Clinical presentation (%, n)*				
Monoarthritis	66.7 (70)	75 (6)	66 (64)	*ns*
Oligoarthritis	18.1 (19)	25 (2)	17.5 (17)	*ns*
Polyarthritis	15.2 (16)	0 (0)	16.5 (16)	*ns*
*Affected joint (%, n)*				
Knee	78.0 (82)	50 (4)	80.4 (78)	*ns*
Hip	6.6 (7)	25 (2)	5.2 (5)	*ns*
Ankle	7.6 (8)	0 (0)	8.2 (8)	*ns*
Shoulder	0.9 (1)	12.5 (1)	0 (0)	*ns*
Elbow	5.7 (6)	12.5 (1)	5.2 (5)	*ns*
Wrist	0.9 (1)	0 (0)	1 (1)	*ns*
Fever (%, n)	39.0 (41)	37.5 (3)	39.2 (38)	*ns*
Duration of symptoms (m ± SD)	6.3 ± 5.8	7.9 ± 5.7	6.2 ± 5.8	*ns*
NSAID (%, *n*)	13.3 (14)	12.5 (1)	13.4 (13)	*ns*
Steroids (%, *n*)	11.4 (12)	25 (2)	9.3 (9)	*ns*
Antibiotic treatment (< 15 days) (%, *n*)	23.8 (25)	37.5 (3)	39.2 (38)	*ns*
CRP (m ± SD)	113.2 ± 94.3	168.5 ± 162.0	108.2 ± 85.4	*ns*
Nucleated cells (m ± SD)	38 297 ± 71 459	66 188 ± 48 056	35 973 ± 72 768	*p* < 0.01
Neutrophils (m ± SD)	32 698 ± 65 323	60 576 ± 43 124	30 300 ± 66 410	*p* < 0.01
Crystals (%, *n*)	61.0 (64)	12.5 (1)	64.9 (63)	*p* < 0.01

*NSAID* non-steroidal anti-inflammatory drug, *CRP* C-reactive protein, *n* number, *m* mean, *SD* standard deviation, *ns* non-significant. Comparison was made between septic and non-septic population

We observed a significantly higher count of nucleated cells in the SP group (66 188 ± 48 056/mm^3^) compared to the NSP group (35,973 ± 72,768/mm^3^, *p* < 0.05). Consistently, the presence of microcrystals was significantly higher in the NSP group (64.9%, *p* < 0.05).

### Trace elements concentration

In the 105 synovial fluids, 16 different trace elements were quantified (Table 2).

**Table 2:** Comparison of metal concentrations between septic and non-septic group

Metals	General population (*n* = 105)	Septic (*n* = 8)	Non-septic (*n* = 97)	*p*-value
Iron *(µg/dl)*	47.0 (41.67–52.4)	84.2 (43.5–124.9)	44.0 (39.3–48.6)	*p* < 0.001
Copper *(µmol/l)*	15.8 (14.9–16.7)	20.5 (14.6–26.4)	15.4 (14.6–16.3)	*p* < 0.05
Zinc *(µmol/l)*	11.3 (10.3–12.4)	20.4 (12.1–28.8)	10.6 (9.7–11.4)	*p* < 0.005
Manganese *(µg/l)*	1.28 (1.19–1.38)	1.89 (1.01–2.77)	1.23 (1.15–1.32)	*p* < 0.05
Magnesium *(mmol/l)*	1.22 (1.18–1.27)	1.52 (1.13–1.92)	1.20 (1.16–1.24)	*p* < 0.01
Selenium *(µg/l)*	47.0 (42.2–51.9)	59.7 (41.1–78.3)	46.1 (41.0–51.2)	*p* < 0.05
Vanadium *(µg/l)*	0.97 (0.95–0.98)	0.96 (0.87–1.06)	0.97 (0.95–0.98)	*ns*
Rubidium *(µg/l)*	249.3 (232.9–265.8)	355.6 (216.1–495.1)	240.6 (226.6–254.5)	*p* < 0.005
Strontium *(µg/l)*	49.6 (40.5–58.7)	29.4 (22.2–36.6)	51.2 (41.4–61.0)	*p* < 0.05
Cobalt *(µg/l)*	0.78 (0.76–0.80)	0.75 (0.69–0.80)	0.78 (0.76–0.81)	*ns*
Lithium *(µg/l)*	8.27 (4.27–12.26)	2.36 (1.29–3.43)	8.75 (4.44–13.07)	*ns*
Nickel *(µg/l)*	3.24 (2.19–4.30)	2.77 (0.15–5.38)	3.3 (2.15–4.41)	*ns*
Chrome *(µg/l)*	2.16 (1.49–2.84)	2.11 (1.23–2.99)	2.17 (1.43–2.90)	*ns*
Zn/Sr ratio	21.3 (18.3–24.3)	47.6 (27.9–67.3)	19.1 (16.6–21.7)	*p* < 0.001
Cu/Zn ratio	1.56 (1.45–1.67)	1.09 (0.84–1.35)	1.60 (1.48–1.71)	p < 0.01

*Zn* zinc, *Cu* copper, *Sr* strontium, Concentrations are presented as mean with a 95% confidence interval, *ns* non-significant. Comparison was made between septic and non-septic population

Septic patients (SP) had significantly higher iron concentrations compared to the non-septic patients (NSP) group (84.2 ± 48.7 vs 44.0 ± 23.13 µg/dl; *p* < 0.001).

Zinc concentration was markedly elevated in the SP group than the NSP group (20.4 ± 10.0 vs 10.57 ± 4.15 µmol/l; *p* < 0.005).

Copper concentration was moderately increased in the SP group, but with a marked drop in the copper-to-zinc ratio. Rubidium and titanium were also substantially greater in the SP group compared to the NSP group.

A significant increase in manganese and selenium concentrations was observed in the SP group compared to the NSP group.

The only metal decreased was strontium in the septic context, leading to a significant difference between the two groups based on the zinc/strontium ratio.

There was no significant variation of vanadium, cobalt, lithium, nickel, and chrome concentration between the two groups. Aluminum and arsenic were also evaluated, but their results were too variable to draw conclusions.

There was no significant difference according to sex for any of the measured metals.

### Diagnosis performance of metal concentration

**Fig. 2:**
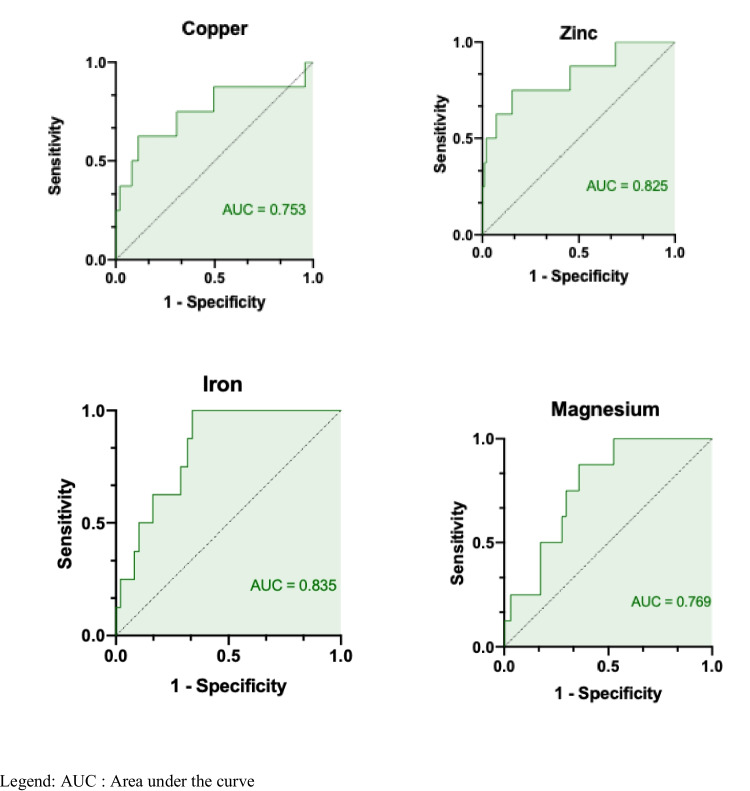
ROC curves illustrating the diagnostic performance. AUC, area under the curve

We then evaluated the diagnosis performance of metals dosage in Table 3 and Fig. 2. ROC curve analysis revealed an interesting discriminative ability of Iron with AUC value of 0.835 (84%, CI 0.732–0.938; *p* < 0.005), and zinc with AUC value of 0.825 (83%; CI 0.653–0.996; *p* < 0.005).

**Table 3:** Diagnostic performance of trace elements

Variable	AUC (CI)	*p*-value
Iron	**0.835** (0.732–0.938)	< 0.005
Copper	**0.753** (0.532–0.974)	< 0.05
Zinc	**0.825** (0.653–0.996)	< 0.005
Manganese	**0.760** (0.596–0.924)	< 0.05
Magnesium	**0.769** (0.641–0.897)	< 0.05
Selenium	**0.756** (0.596–0.917)	< 0.05
Rubidium	**0.794** (0.646–0.941)	< 0.005
Strontium	**0.709** (0.564–0.854)	< 0.05
White blood cell count	**0.788** (0.662–0.914)	< 0.01
Neutrophils count	**0.810** (0.695–0.925)	< 0.01
Cu/Zn ratio	**0.785** (0.641–0.929)	< 0.01
Zn/Sr ratio	**0.880** (0.760–1.000)	< 0.001

*Zn* zinc, *Cu* copper, *Sr* strontium, *AUC* area under the curve

AUC value of rubidium alone (0.794; 79%; CI 0.646–0.941; *p* < 0.005) and the copper/zinc ratio (0.785, 79%; CI 0.641–0.929; *p* < 0.01) showed also a good diagnostic performance.

An acceptable level of accuracy was observed for copper (75%; CI 0.532–0.974; *p* < 0.05), manganese (76%, CI 0.596–0.924; *p* < 0.05), magnesium (77%; CI 0.641–0.897; *p* < 0.05), and strontium (71%; CI 0.564–0.854; *p* < 0.05).

Interestingly, zinc/strontium ratio has an AUC value of 0.880 (85%, CI. 0.760–1.000; *p* < 0.001), which suggested a good discriminative ability as a promising parameter for further analysis.

### Sensitivity analysis

We then conducted sensitivity analyses to identify thresholds that could be useful for diagnosis (Supplementary Table S2). The values that achieved 100% sensitivity exhibited very low specificity, often below 50%, making them insufficient on their own to rule out septic arthritis. The only exception was the iron threshold of 48 µg/dL, which achieves 100% sensitivity but with a specificity of 66%. Using Youden’s index, we identified more accurate thresholds, such as 13.5 µmol/L for zinc (Se 75%, Spe 85%), 1.3 mmol/L for magnesium (Se 75%, Spe 70%), 278 µg/L for rubidium (Se 75%, Spe 79%), and 25.32 for zinc/strontium ratio (Se 88%, Spe 80%).

### Logistic regression analysis

We used the thresholds from the sensitivity analysis to determine the corresponding ORs and thus measure the association between the variable and the diagnosis of septic arthritis in Supplementary Figure S1 and S2. The sensitivity analysis approach may amplify apparent associations in the context of a small number of events, while still remaining close to clinical reality. The odds ratios reported should therefore be interpreted as exploratory estimates.

## Discussion

In our study, we observed a significant variation of metals concentrations in septic arthritis compared to non-septic arthritis. Iron, zinc, magnesium, rubidium, copper, manganese, and selenium concentrations in the synovial fluid were markedly increased during acute septic arthritis. Among all metals, only strontium exhibited a statistically significant decrease in the same context. Logistic regression found a positive association between iron, copper, manganese, zinc, magnesium, and rubidium SF levels and septic arthritis.

Trace elements are essential cofactors in numerous enzymatic processes in both humans and microorganisms. Their homeostasis is tightly adjusted to avoid deficiency or toxicity, leading to the concept of nutritional immunity: this concept refers to the ability of the host to sequester trace elements to inhibit the pathogenicity of invading microorganisms while regulating its own immune cell function [29]. It led to significant interest to study variation of metal trace elements in various biological fluids during infection, as these fluctuations may reflect the presence of an infectious process. Although trace element analysis is predominantly performed on blood samples, studies have investigated their levels in other biological fluids such as urine or bronchoalveolar lavages [23–26].

In our population, the prevalence of septic arthritis was initially 10%. After excluding three hemolyzed samples, this rate decreased to 8%, which remained within the lower range of expected prevalence of septic arthritis [4]. Several types of bacteria were represented, with a predominance of Gram-positive cocci, which is consistent with the epidemiology typically observed in septic arthritis.

First, we found that iron was significantly increased in septic synovial fluid. While other studies associated iron-regulating protein activity with synovial inflammation [30, 31], they were limited to osteoarthritic knee-joint effusions or IRD [18]. However, iron is the most widely used trace element in biological processes, with elevated concentrations also reported in other infected fluids. Pathogens such as *Escherichia coli* and *Pseudomonas aeruginosa* are able to use siderophores, iron-chelating molecules produced by bacteria [20] to capture iron from their environment, to ensure their survival and virulence, especially in iron-limited environments like infected tissues. This property is used in therapy with the cephalosporin called Cefiderocol, which targets bacteria via siderophore-mediated iron transport, allowing it to limit some bacterial resistance mechanisms. Higher iron concentration is also observed in respiratory samples such as expectorations and Bronchoalveolar Lavage Fluid in cystic fibrosis patients for *Pseudomonas aeruginosa* or *Mycoplasma bovis* [23, 32] reinforcing our results.

Then, we observed a significant increase in Zinc concentration in septic synovial fluid, with interesting diagnostic performance. In the literature, there are discrepancies regarding concentration differences in purely inflammatory fluids, such as in RA [17, 18]. Zinc is crucial in structural processes due to its cofactor function in various enzyme activities, involved in protein and DNA metabolism [33]. The connection between zinc and immune function is well-established, but the precise mechanisms remain unclear. Interestingly, zinc has direct antimicrobial properties, notably against *Staphylococcus aureus* infection, the most commonly isolated pathogen in septic arthritis documentation [34]. Zinc and manganese are also key targets of calprotectin, a promising biomarker for septic arthritis and periprosthetic joint infections [35–37]. Nevertheless, our results did not show significant differences in the Zn/Mn ratio, compared to findings in *Streptococcus pneumoniae* infection models [34].

The dosage of copper appeared to be moderately elevated in septic fluid. Excessive copper concentration becomes highly toxic with production of reactive hydroxyl radicals. In urine samples, metal profiles vary depending on the type and location of the infection, with a notable increase in copper excretion in urinary tract infections [25] but also of the ceruloplasmin activity. Conversely, there is a marked decrease in copper levels in urine in case of SARS-CoV-2 viral pulmonary infection. Although moderately increased in our study, copper levels appeared closely correlated with zinc, as observed in pleural effusions where zinc and copper are elevated in exudates compared to transudates [23]. The copper/zinc ratio is also associated with inflammation and considered as an effective tool to identify high-risk individuals for infection especially in pneumonia.

Interestingly, strontium level was decreased in septic fluids, with a strong discriminative power for the zinc/strontium ratio. Though strontium is known to support bone formation through osteoblast activity, and decreased in RA [17], its relationship with infection remains unclear.

Magnesium, selenium, manganese, and rubidium concentrations were also significantly increased in septic fluids in our results. Magnesium, essential for membrane and ribosome stability, plays a key role in bacterial survival, as seen in digestive infections [38]. Selenium and manganese are involved in oxidative stress responses and are modulated differently in non-septic conditions such as RA [18]. Interestingly, elevated Selenium is observed in infected breast milk [39]. Rubidium, a potassium analog, is elevated in other infections such as thoracic empyema and brain abscesses [40].

This study had some limitations. The most important one is the very small number of septic arthritis cases (*n* = 8), limiting the reliability of all statistical analyses performed, including ROC curve analysis and logistic regression. Also, the simultaneous screening of trace element was deliberate, as the primary aim of this exploratory study was to identify candidate metals of interest for future targeted and adequately powered prospective studies. Results should therefore be interpreted as hypothesis-generating rather than conclusive. Although the non-septic group is heterogeneous, preliminary analyses did not reveal significant differences in trace element concentrations between microcrystalline and inflammatory subgroups. Since this is a retrospective study based on a cohort where a significant amount of synovial fluid had already been utilized, we predominantly had knee joint fluids, as a sufficient volume was required for analysis. Due to the retrospective nature of the study, plasma assays could not be performed, limiting the possibility to correlate serum and synovial levels. Data on iron, zinc, or other trace element supplementation were not systematically recorded and could not be retrieved. This represents a potential source of bias that should be addressed in future prospective studies. We used the ICP-MS technique to quantify trace elements in the synovial fluid. This technique has high accuracy and sensitivity, but poor accessibility and requires a long time to obtain results. However, iron or magnesium can be potentially measured in routine biochemistry by classical colorimetry analysis, with delays depending on the center in standard laboratory practice.

Our study had several strengths. To our knowledge, this is the first study exploring the measurement of metals in synovial fluid in a septic context. We decided to exclude hemolyzed fluids in order to limit interactions with trace element concentration such as iron or zinc. We also had to exclude two samples with high selenium concentrations. Selenium levels were excessively increased due to an MRI contrast injection within the previous 24 h. In light of the various reasons outlined previously, it seems crucial to confirm these results with a multicenter study involving a larger cohort. It may also be interesting to investigate the variability related to different pathogens.

## Conclusion

In conclusion, this exploratory study suggests that synovial fluid metallomic profiling may help differentiate septic from non-septic arthritis, with iron, zinc, copper, manganese, magnesium, rubidium, and strontium showing promising preliminary discriminative value. However, given the small number of septic cases, the retrospective single-center design, and the methodological limitations detailed above, these findings should be considered hypothesis-generating only. Prospective validation in larger, multicenter cohorts is necessary before any clinical application can be considered.

## Supplementary Information

Below is the link to the electronic supplementary material.

Supplementary Material 1 (DOCX 77.5 KB)

## Data Availability

The data supporting the findings if this study are available from the corresponding author upon request.
