# Supplementary material for: Investigating synovial trace elements as diagnostic markers in acute septic arthritis: an exploratory study
Source: Clin Rheumatol. 2026 May 22;45(7):4615–23. doi: 10.1007/s10067-026-08171-2 (PMC13342201; doi:10.1007/s10067-026-08171-2)
Supplement: Supplementary file 1 — Supplementary Material 1 (DOCX 77.5 KB) [file 10067_2026_8171_MOESM1_ESM.docx]

***Supplementary Figure S1: Forest plot of OR (95%CI with log scale)***


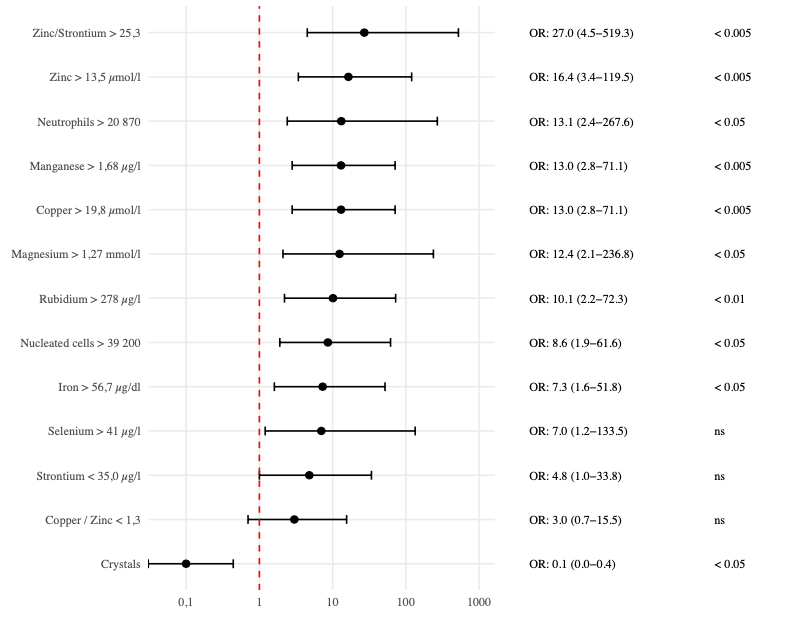


Legend: OR: Odd Ratio; 95% confidence interval

***Supplementary Table S1: Association of variables with the septic diagnosis***

| Variable | OR | p-value |
| --- | --- | --- |
| Iron > 56,7 µg/dl | **7,3** (1,6 - 51,8) | < 0,05 |
| Copper > 19,8 µmol/l | **13,0** (2,8 - 71,1) | < 0,005 |
| Zinc > 13,5 µmol/l | **16,4** (3,4 - 119,5) | < 0,005 |
| Manganese > 1,68 µg/l | **13,0** (2,8 - 71,1) | < 0,005 |
| Magnesium > 1,27 mmol/l | **12,4** (2,1 - 236,8) | < 0,05 |
| Selenium > 41 µg/l | **7,0** (1,2 -133,5) | *ns* |
| Rubidium > 278 µg/l | **10,1** (2,2 - 72,3) | < 0,01 |
| Strontium < 35,0 µg/l | **4,8** (1,0 - 33,8) | *ns* |
| Zn/Sr > 25,3 | **27,0** (4,5 - 519,3) | < 0,005 |
| Cu / Zn < 1,3 | **3,0** (0,7 - 15,5) | *ns* |
| Nucleated cells > 39 200 | **8,6** (1,9 - 61,6) | < 0,05 |
| Neutrophils > 20 870 | **13,1** (2,4 - 267,6) | < 0,05 |
| Crystals | **0,1** (0,0 - 0,44) | < 0,05 |

Legend: OR: Odd Ratio; 95% confidence interval

***Supplementary Table S2 : Sensitivity Analysis of Concentration Thresholds***

| Metals | Threshold | Se | Spe | LR+ | LR- | Youden index |
| --- | --- | --- | --- | --- | --- | --- |
| Iron (µg/dl) | **48,00** | 1,0 (0.68 - 1.00) | 0,66 (0,56 - 0,75) | 2,94 | 0,00 | 0,66 |
|  | **50,30** | 0,88 (0,53 - 0,99) | 0,68 (0,58 - 0, 76) | 2,75 | 0,18 | 0,555 |
|  | **56,70** | 0,75 (0,41 - 0,96) | 0,71 (0,61 - 0,79) | 2,59 | 0,35 | 0,461 |
| Copper (µmol/l) | **8,30** | 1,0 (0,68 - 1,00) | 0,04 (0,02 - 0,1) | 1,04 | 0,00 | 0,041 |
|  | **15,20** | 0,88 (0,53 - 0,99) | 0,51 (0,41 - 0,60) | 1,80 | 0,24 | 0,375 |
|  | **19,80** | 0,63 (0.31 - 0.86) | 0,89 (0,81 - 0,94) | 5,73 | 0,42 | 0,512 |
| Zinc (µmol/l) | **8,70** | 1,0 (0,68 - 1,00) | 0,31 (0,23 - 0,41) | 1,45 | 0,00 | 0,309 |
|  | **10,30** | 0,88 (0,53 - 0,99) | 0,55 (0,45 - 0, 64) | 1,96 | 0,22 | 0,421 |
|  | **13,50** | 0,75 (0,41 - 0,96) | 0,85 (0,76 - 0,90) | 5,00 | 0,29 | 0,595 |
| Manganese (µg/l) | **1,04** | 1,0 (0,68 - 1,00) | 0,38 (0,29 - 0,48) | 1,61 | 0,00 | 0,381 |
|  | **1,09** | 0,88 (0,53 - 0,99) | 0,47 (0,38 - 0,57) | 1,66 | 0,26 | 0,349 |
|  | **1,68** | 0,63 (0,31 - 0,86) | 0,89 (0,81 - 0,94) | 5,73 | 0,42 | 0,512 |
| Magnesium (mmol/) | **1,20** | 1,0 (0,68 - 1,00) | 0,47 (0,38 - 0,57) | 1,89 | 0,00 | 0,474 |
|  | **1,27** | 0,88 (0,53 - 0,99) | 0,64 (0,54 - 0,73) | 2,44 | 0,19 | 0,514 |
|  | **1,31** | 0,75 (0,41 - 0,96) | 0,70 (0,60 - 0,78) | 2,50 | 0,36 | 0,451 |
| Selenium (µg/l) | **39,16** | 1,0 (0,68 - 1,00) | 0,42 (0,32 - 0,52) | 1,72 | 0,00 | 0,417 |
|  | **41,00** | 0,88 (0,49 - 0,99) | 0,5 (0,40 - 0,60) | 1,76 | 0,24 | 0,357 |
|  | **43,96** | 0,71 (0,36 - 0,95) | 0,58 (0,48 - 0,68) | 1,69 | 0,50 | 0,298 |
| Rubidium (µg/l) | **210,8** | 1,00 (0,68 - 1,00) | 0,35 (0,26 - 0,45) | 1,54 | 0,00 | 0,351 |
|  | **252,50** | 0,88 (0,53 - 0,99) | 0,64 (0,54 - 0,73) | 2,44 | 0,19 | 0,514 |
|  | **278,30** | 0,75 (0,41 - 0,96) | 0,79 (0,71 - 0,86) | 3,57 | 0,32 | 0,544 |
| Strontium (µg/l) | **41,87** | 1,00 (0,68 - 1,00) | 0,42 (0,33 - 0,52) | 1,72 | 0,00 | 0,423 |
|  | **41,08** | 0,88 (0,53 - 0,99) | 0,44 (0,35 - 0,54) | 1,57 | 0,27 | 0,318 |
|  | **34,97** | 0,75 (0,41 - 0,96) | 0,62 (0,52 - 0,71) | 1,97 | 0,40 | 0,369 |
| Zinc/Strontium ratio | **16,18** | 1,00 (0,68 - 1,00) | 0,47 (0,38 - 0,57) | 1,89 | 0,00 | 0,474 |
|  | **25,32** | 0,88 (0,53 - 0,99) | 0,80 (0,71 - 0, 87) | 4,40 | 0,15 | 0,679 |
| Copper / Zinc ratio | **1,53** | 1,00 (0,68 - 1,00) | 0,46 (0,37 - 0,56) | 1,85 | 0,00 | 0,464 |
|  | **1,47** | 0,88 (0,53 - 0,99) | 0,53 (0,43 - 0,62) | 1,87 | 0,23 | 0,401 |
|  | **1,30** | 0,75 (0,41 - 0,96) | 0,66 (0,55 - 0,74) | 2,21 | 0,38 | 0,275 |
| Nucleated cells | **15 550** | 1,0 (0,66 - 1,00) | 0,49 (0,39 - 0,59) | 1,96 | 0,00 | 0,49 |
|  | **23 350** | 0,88 (0,53 - 0,99) | 0,61 (0,51 - 0,71) | 2,26 | 0,20 | 0,49 |
|  | **39 200** | 0,75 (0,41 - 0,96) | 0,75 (0,66 - 0,83) | 3,00 | 0,33 | 0,5 |
| Neutrophils | **14 174** | 1,0 (0,68 - 1) | 0,55 (0,45 - 0,66) | 2,22 | 0,00 | 0,548 |
|  | **20 870** | 0,88 (0,53 - 0,99) | 0,66 (0,55 - 0,74) | 2,59 | 0,18 | 0,531 |
|  | **35 268** | 0,75 (0,41 - 0,96) | 0,76 (0,67 -0,84) | 3,13 | 0,33 | 0,513 |

Legend: 95% confidence interval, Se: Sensitivity ; Spe: Specificity; LR+: Positive Likelihood Ratio; LR-: Negative Likelihood Ratio; Copper/Zinc ratio and strontium concentrations are negatively correlated with septic diagnosis.
